# Supplementary material for: The Association of Prostate Cancer and Urinary Tract Infections: A New Perspective of Prostate Cancer Pathogenesis
Source: Medicina (Kaunas). 2023 Mar 1;59(3):483. doi: 10.3390/medicina59030483 (PMC10056160; doi:10.3390/medicina59030483)
Supplement: Supplementary file 1 [file medicina-59-00483-s001.zip › medicina-2213189-supplementary.pdf]

medicina MDPI

Table S1. Baseline characteristics of the patients with or without prostate cancer after propensity score matched.

Variables	Without prostate cancer N=2548		With prostate cancer N=2548		
	n	%	n	%	p-value
Age, years					0.97
20-64	473	18.6	474	18.6	
>=65	2075	81.4	2074	81.4	
Mean±SD [a]					
Comorbidity					
Hypertension	1617	63.5	1623	63.7	0.86
Diabetes	667	26.2	656	25.8	0.73
Hyperlipidemia	906	35.6	906	35.6	0.99
COPD	702	27.6	718	28.2	0.62
CKD	341	13.4	356	14.0	0.54
Treatments					
Radiotherapy			1025	40.2	
Chemical therapy			338	13.3	
Radical prostatectomy			1913	75.1	
Androgen deprivation therapy			1128	44.3	

[a] t-test; Chi-square test.

Abbreviations: COPD, chronic obstructive pulmonary disease; CKD, chronic kidney disease

Table S2. Comparisons of the incidence of urinary tract infections, prostatitis, cystitis, and pyelonephritis between the patients with and without prostate cancer after propensity score matched.

	Without prostate cancer			With prostate cancer			Crude		Adjusted	
Variable	Event	Person-Year	IR	Event	Person-Year	IR	HR (95% CI)	P-value	HR (95% CI)	P-value
Overall	534	15013	35.6	715	12450	57.4	1.59(1.42, 1.78)	<0.001	1.58(1.41, 1.77)	<0.001
Urinary tract infection	524	15052	34.8	693	12534	55.3	1.56(1.39, 1.75)	<0.001	1.55(1.39, 1.74)	<0.001
Recurrent UTIs	302		20.1	405		32.3	1.58(1.36, 1.84)	<0.001	1.57(1.35, 1.83)	<0.001
Prostatitis	11	17144	0.64	20	14899	1.34	2.10(1.01, 4.38)	0.048	2.10(1.00, 4.38)	0.049
Cystitis	12	17124	0.70	37	14799	2.50	3.60(1.87, 6.90)	0.001	3.57(1.86, 6.86)	0.001
Pyelonephritis	19	17094	1.11	38	14897	2.55	2.33(1.34, 4.03)	0.003	2.29(1.32, 3.97)	0.003

Abbreviations: IR, incidence rate, per 1000 person-years; HR, hazard ratio; CI, confidence interval. Adjusted HR: adjusted for age, sex, and comorbidities in Cox proportional hazards regression.

Table S3. Cox proportional hazard regression analysis for the risk of urinary tract infection with interaction of Radiotherapy and Androgen deprivation therapy among prostate cancer patients.

Radiotherapy	Androgen deprivation therapy	N	Event	IR	Crude HR (95% CI)	Adjusted HR (95% CI)
No	No	1043	353	69.5	1(Ref)	1(Ref)
Yes	No	497	152	50.1	0.74(0.61, 0.90)	0.71(0.59, 0.86)
No	Yes	606	160	61.0	0.85(0.70, 1.02)	0.84(0.70, 1.02)
Yes	Yes	609	119	43.0	0.60(0.49, 0.74)	0.61(0.49, 0.75)

HR, hazard ratio; CI, confidence interval; Adjusted HR: adjusted for age, sex, comorbidities, and other medications in Cox proportional hazards regression; P for interaction=0.78
